# A Comprehensive Analysis of COVID-19 Impact in Latin America

**DOI:** 10.21203/rs.3.rs-141245/v1

**Published:** 2021-01-08

**Authors:** Hassan Ashktorab, Antonio Pizuomo, Nora Alma Fierro González, Edgar Daniel Copado Villagrana, María Evangelina Herrera-Solís, Graciela Cardenas, Daniela Zavala-Alvarez, Gholamreza Oskrochi, Eyitope Awoyemi, Folake Adeleye, Maryam Mehdipour Dalivand, Adeyinka O Laiyemo, Edward E. Lee, Farshad Aduli, Zaki A Sherif, Hassan Brim

**Affiliations:** Howard University College of Medicine; La Universidad del Zulia; National Autonomous University of Mexico; Mexican Social Security Institute; Mexican Social Security Institute; Mexican Social Security Institute: Instituto Mexicano del Seguro Social; Institute of Neurology and Neurosurgery: Instituto de Neurologia y Neurocirugia; American University of the Middleeast; Howard University College of Medicine; Howard University College of Medicine; Howard University College of Medicine; Howard University Hospital; Howard University College of Medicine; Howard University Hospital; Howard University College of Medicine; Howard University College of Medicine

**Keywords:** coronavirus disease-19, pandemic, Gastrointestinal manifestation, diarrhea

## Abstract

**Background:**

Latin America has now become the epicenter of the global coronavirus disease 2019 (COVID-19) pandemic. In the ongoing COVID -19 pandemic, a profound burden of SARS-COV-2 infection has been reported in Latin America. In the present study, we aim to determine the profiles that are associated with this disease in Latin America. We analyzed symptoms, morbidities and gastrointestinal (GI) manifestations by country.

**Methods:**

We analyzed data from SARS-CoV-2 positive patients evaluated at healthcare centers and hospitals of 8 Latin American countries including Brazil, Peru, Mexico, Argentina, Colombia, Venezuela, Ecuador, and Bolivia between March 1 and July 30, 2020. These countries consist of a total population that exceeds 519 million. Demographics, comorbidities and clinical symptoms were collected. Statistical descriptive analysis and correlation analyses of symptoms, comorbidities and lethality were performed.

**Results:**

A total of 728,282 patients tested positive for COVID-19 across all the 8 Latin American countries. Of these, 52.6% were female. The average age was 48.4 years. Peru had the oldest cohort with 56.8 years old and highest rate of females (56.8%) while Chile had the youngest cohort (39 years old). Venezuela had the highest male prevalence (56.7%). Most common symptoms were cough with 60.1% (Bolivia had the highest rate 78%), fatigue/tiredness with 52.0%, sore throat with 50.3%, and fever with 44.2%. Bolivia had fever as the top symptom (83.3%). GI symptoms including diarrhea (highest in Mexico with 22.9%), nausea, vomiting, and abdominal pain were not associated with higher mortality.

Hypertension was among the top (12.1%) comorbidities followed by diabetes with 8.3% and obesity 4.5%. In multivariable analyses, the leading and significant comorbidities were hypertension (r=0.83, p=0.02), diabetes (r=0.91, p=0.01), and obesity (r=0.86, p=0.03). Asthma (r=0.37, p=0.54) and increasing age (0.13 p=0.81) were not independently associated with higher mortality. Lethality was highest in Mexico (16.6%) and lowest in Venezuela (0.9%) among the analyzed cohorts.

**Conclusion:**

Nearly, 10.5%–53% of patients with COVID-19 have GI manifestations. Differential clinical symptoms were associated with COVID-19 in Latin America countries. Metabolic syndrome components were the main comorbidities associated with poor outcome. Country-specific management and prevention plans are needed. Country-specific management and prevention plans can be established from this meta-analysis.

## Background

Latin America has recently experienced unique and unprecedented challenges with emerging viral diseases including Chikungunya and Zika, that were endemic to some regions [1,2]. However, the Coronavirus Disease 2019 (COVID-19) may be the most lethal to date [3,4]. On December 31, 2019, in the city of Wuhan in Hubei province of China [4], the first report was made of a pneumonia-like cluster of illnesses whose common symptoms included fever, dry cough, and in some cases severely compromised breathing (i.e. dyspnea). The initial report indicated that most of the patients either worked at, or visited a wet wholesale seafood market called Huanan Seafood Wholesale Market where live wild animals and meat were regularly traded and sold [5]. On January 7, according to the World Health Organization (WHO), Chinese officials announced they had identified a new virus named 2019-nCoV and was identified as belonging to the coronavirus family, which includes SARS and the common cold. On January 10, 2020, Chinese scientists published the entire genome of this new pathogen [5]. Although there are currently circulating respiratory coronaviruses of the family Coronaviridae, genus Betacorona virus, in the human population, this novel strain was more contagious, virulent and stealthier. WHO officially named the disease COVID-19 (Coronavirus Disease 2019) [5]. On January 30, WHO declared the outbreak a Public Health Emergency of International Concern (pandemic) and the virus SARS-CoV-2 (Severe Acute Respiratory Syndrome Coronavirus-2) as the successor to SARS-CoV-1 on March 11, 2020 [6]. As of September 5th, 2020, the disease has killed more than 867,000 people and infected over 26 million, according to the evolving data compiled by Johns Hopkins University[7]. While the majority of infected patients did not succumb to the disease, short- and long-term effects are yet to be assessed, especially as it pertains to non-lethal symptoms’ complications.

The burden of COVID-19 in Latin America is enormous (Fig. 1Suppl). Given the ongoing and continuous transmission, the high rates of subclinical infections, inconsistent and insufficient diagnostic testing from country to country, there are differences in attribution of infected cases and cause of death. Compared to the US, the high number of COVID-19 in Latin American nations is especially troubling because of the already fragile economies compounded by months of strict lockdowns and weaker healthcare systems that may provoke adverse social and political turmoil in the future. Latin American countries also harbor densely populated cities like Mexico City with 8.918,653 habitants [8], Sao Paulo with 11,253,503 habitants [9], Buenos Aires with 2,891,08 habitants [10] and urban slums that are hit with extreme poverty and overcrowded conditions, fertile grounds for community transmission of the virus and widespread propagation of the infection [11].

Brazil, the Latin American country hardest hit by the pandemic, may be undertesting with the real figures probably far higher [12]. The contribution to a higher infection and death toll might have come from the participation of millions of tourists in carnivals in the streets of Rio de Janeiro, Sao Paulo, Salvador and other cities which started the celebration on February 21, 2020 [13]. However, the rapid spread of the pandemic and the small number of tests performed in many Latin American countries including Peru, Mexico, Argentina, Columbia and Bolivia make it difficult to estimate the actual number of cases to initiate control measures, resource planning, or benchmarking with neighboring countries.

The confirmed cases of COVID-19 are the most crucial data to understand the evolution of the disease and the symptoms it precipitates. The earliest cases of COVID-19 in China were identified through “pneumonia-like” cases of unknown origin [5]. However, the spread of the infection throughout the world including Latin America has revealed that there are numerous ailments including neurological, cardiovascular, immunological and gastrointestinal that could be caused by SARS-CoV-2 [14].

In this study, we described the clinical characteristics, lethality, and symptoms of the confirmed COVID-19 cases officially registered by countries that make pertinent information publicly available. Special emphasis was made on gastrointestinal symptoms and comorbidities as they relate to lethality of the disease in Latin America.

## Methods

### Search Strategy and Selection Criteria

We conducted a systematic literature search of published articles using electronic databases such as PubMed, OVID, Scopus, Google Scholar, LANCOVID (Latin America research network on COVID), and other resources from official health organizations of countries in Latin America such as Ministries of Health, National Institutes of Hygiene, or Hospitals from January 1 through July 30, 2020. We included the following terms in the search bar: COVID-19, Argentina, Brazil, Chile, Colombia, Ecuador, Mexico, Peru, Venezuela, Uruguay, Paraguay, Hispanic and Latin America or South America. Additionally, we searched for official and non-official press releases of patient and hospital data from government health institutions of the leading COVID-19 virus-infected Latin American countries. We searched leading reference databases, primary published sources and press releases from the various Ministries of Health of the respective Latin American countries. Case characteristics were described, including demographics, exposures, comorbidities and symptoms.

### Selection and Identification of Relevant Literature:

Using the listed inclusion and exclusion criteria, we first sorted the Latin American COVID-19 studies by title and abstract; then we compiled the papers by relevance and conducted a new selection process by a thorough review of the data. Based on detailed insights derived from the most relevant papers, we revised our reference search criteria to obtain more refined papers for our systematic review. We incorporated studies that reported patients’ characteristics and symptoms of interest. From the selected papers, tables were generated for each dataset on Microsoft Excel. These tables included the following information for extracted data of each study: Study author (year, location, hospital or city, state and country), date of the report, location, confirmed cases, deaths, lethality rate, average age, sex, headache (cephalea), cough, myalgias, fever, odynophagia, anosmia, dyspnea, ageusia, diarrhea, thoracic pain, abdominal pain, tachypnea, cyanosis, sore throat, nasal congestion, nausea and vomiting, fatigue or tiredness, joint pain, hypertension, diabetes mellitus, asthma, obesity, cardiovascular disease, chronic cardiopathy, chronic obstructive pulmonary disease, chronic kidney disease, chronic neurological disease, cancer, inflammatory bowel disease, and tuberculosis (Fig. 1).

Inclusion Criteria: The following inclusion criteria were adopted to validate article selection: Confirmed diagnosis of COVID-19 as reported by each country and referenced by WHO or cross-checked via World-Meter or Johns Hopkins University COVID-19 dashboard; No distinction with regard to the number of diagnosed cases; No distinction with regard to sex, age, treatment and outcome. Exclusion Criteria: The following exclusion criteria were adopted to filter out incomplete or ambiguous data: Studies where the cohort was not Latino nor Hispanic. Studies where the cases were not confirmed by RT-PCR, and studies with incomplete symptoms or comorbidities report.

### Statistical Analysis:

The data was used to calculate COVID-19 inpatients from 8 Latin American countries; with Peru (2 studies, 357,681 patients), Chile (306,440 patients), Argentina (36,749 patients), Mexico (2 studies, 3,110 patients), Ecuador (9,468 patients), Bolivia (107 patients), Venezuela (4,014 patients), and Brazil (10,713 patients). A total of 728,282 patients with a collective death toll of 25,008 (3.4%). The common symptoms and comorbidities were combined and analyzed by weighted analysis methods where applicable. Correlation coefficients were calculated together with regression analysis to establish associations between comorbidities and lethality. The effect of symptoms was reported using weighted analysis where weights were related to the size of the reported study. SPSS (SPSS Inc., Chicago, IL, USA) was used for this analysis.

## Results

### Demographic and clinical characteristics

There were 728,282 confirmed cases in our study from 8 countries (Table 1). These countries are: Peru, Ecuador, Bolivia, Mexico, Chile, Argentina, Venezuela and Brazil. As for the characteristics of each cohort, there was no discrimination between patients who were hospitalized versus those treated at ambulatory services and not admitted (outpatients). The largest cohort was from Chile with 306,440 (Table 1) confirmed cases and 8,580 deaths, second was Argentina with 57,729 patients and 1,196 deaths. Overall, the number of deaths for the countries were 25,004 patients. For case fatality, only Chile and Argentina were the ones that reported highest numbers of deaths. No treatment data was reported by any of the Latin American authors or public health organizations.

### Adults < 50 were the most common infected group in Latin America

**Figure 2:**
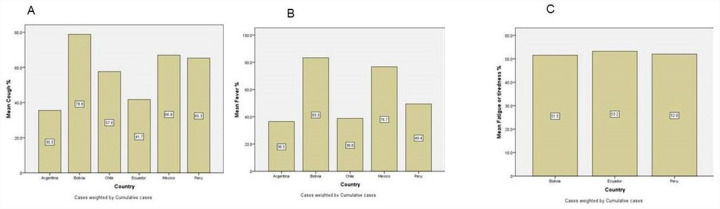
Comparison of (%) mean A) Cough, B) Fever symptom, and C) Fatigue and tiredness, in selected studies from different countries.

The average age for this Latin American cohort was 48.4 years (Table 1). There were age differences in this cohort of COVID-19 patients. Ages ranged from 39 to 56.8 years. The cohort from Peru was the oldest with 56.8 years old average, in second Mexico with 47.4 years old and in third place Brazil with 44.8. The youngest cohort was from Chile with an average age of 39 years (Fig. 2 Suppl.). The 8 cohorts have uniform age distribution (KS test with p = 0.41)

### Sex differences in patients diagnosed with COVID-19 in Latin America

The distribution between males and females was 47.3% versus 52.6%, respectively. The percentage of females infected with COVID-19 was highest (56.8%) in Peru and Argentina (54%) while other Latin American coun-tries showed lower percentages with Venezuela reporting the lowest (43.3%) (Fig. 3 Suppl.). The cohort from Venezuela reported the most males with 56.7%, while other countries had comparable rates with Ecuador (55.4%), Brazil (55.2), Mexico (53.1%), Chile (51.6%), Bolivia (51.4%). Peru with 43.2% reported the lowest percentage of males infected with COVID 19. However, the 8 cohorts had comparable overall sex distributions (KS test p = 0.26).

### Cough and fatigue were the top symptoms in Latin American COVID-19 patients

According to the combined overall weighted average, the most common symptoms reported in the 8 Latin American countries for positive cases of SARS-CoV-2 infection were cough with 60.1%, fatigue or tiredness with 52.0%, sore throat with 50.3%, and fever with 44.2% (Table 1). Bolivia was the country that reported the highest positivity for cough (Fig. 2A) 78.8% and fever (Fig. 2B); at least 12% more than Mexico in second place. Ecuador reported the highest prevalence for fatigue followed by Peru and Bolivia with 52% and 51.5%, respectively (Fig. 2C).

### Fever was not one of the main symptoms in Latin American COVID-19 patients

While fever is probably one of the most common symptoms for COVID-19, that was not the case for the population we studied in Latin America. Fever was the 4th most common symptom with a mean of 44% and 3rd was Sore Throat (50.3%), dry cough and fatigue (Table 1). In our report, Bolivia was the region that reported 83.3% of fever, followed by Mexico with 76.6% and then at a much lower rate Peru with 49.4% (Fig. 2B).

### Gastrointestinal manifestations were highest in Mexico among Latin America COVID-19 patients

Our cohort of patients reported a different array of gastrointestinal symptoms including diarrhea, abdominal pain, nausea, and vomiting. Diarrhea was the most reported symptom being present in 11.5% of patients (Table 3). There was no characterization of the type of diarrhea, whether or not it contained blood; mucus or pus. It is important to mention that only Argentina, Chile, Mexico and Peru (Fig. 3) reported diarrhea on their symptoms and Mexico was the country with the highest prevalence (22.9%), followed by Peru with 13.6%. Diarrhea was followed by nausea and vomiting in 7.2% of patients and lastly abdominal pain in 4.3% in the whole group analysis (Table 2). As for gastrointestinal diseases, the prevalence of chronic hepatic disease was 0.31% in positive cases and as such the least common comorbidity (Table 2).

### Hypertension was among the top comorbidities in Latin America COVID-19 patients

Among comorbidities, the reports showed that hypertension was the most common with an average of 12.1% followed by diabetes with 8.3% and obesity with 4.5% (Table 2). Overall, 12.1% of the cohort had at least one comorbidity. It’s also important to note that patients can have multiple symptoms and comorbidities. Differences for comorbidities were present among different countries. Mexico was the country with the highest prevalence of hypertension with almost 28.8% followed by Chile with 16% (Fig. 4A) and for diabetes as well (Fig. 4C). Mexico reported the highest prevalence with 20.6% followed by Chile with 8%. As for obesity (Fig. 4B), Mexico had the highest prevalence with 25.6% followed by Brazil with a 23.7% of the total of their cohorts. As such, the cohort from Mexico was the most hypertensive, diabetic and obese of all 8 countries.

### Lethality was highest in Mexico among Latin American countries

We analyzed the lethality rate for the 8 countries, and there are some important differences between them. Lethality is defined as the number of deaths as end result of COVID-19 over number of positive COVID-19 (x100) (also known as Case Fatality Rate) in the population. The overall weighted average for the total population of our study (Table 1 Suppl.) showed that the lethality rate was 3.4%. In relation to the stratification of the lethality rate per country (Fig. 8), there were mixed results, with Mexico reporting a lethality of 16.6%, followed by Brazil with 7.6% and then Bolivia with 5.6%. Venezuela reported the lowest lethality rate with 0.9%.

The variables result related to lethality rate (Fig. 5) showed that Mexico reported the highest rate with 16.7%, followed by Brazil with 7.6% and Bolivia with 5.6%. Venezuela reported the lowest lethality rate with just 0.95%. Overall, there were several differences in the reported lethality rates.

### Association of lethality and comorbidities in COVID-19 patients

We explored the association of lethality rate as a dependent variable in univariate and multivariate analyses with several independent variables such as obesity (Table 2 Suppl), hypertension, diabetes, and asthma (Table 2 Suppl.). Each of these analyses was statistically significant except for asthma (Table 2 Suppl.). This data shows that hypertension, diabetes and obesity are associated with lethality in COVID-19 patients in Latin America. A multiple regression analysis shows that the most influential factor is obesity followed by hypertension and diabetes.

### Lethality and GI manifestations in COVID-19 patients in Latin America

We explored the association of lethality rate as a dependent variable in univariate and multivariate analyses with several independent variables such as GI symptoms. None of the symptoms including diarrhea, abdominal pain, nausea/vomiting individually or collectively was associated with lethality in Latin America.

## Discussion

COVID-19 is a consequence of infection with the SARS-CoV-2 virus. It is well known that previous Coronavirus infections attack mainly the respiratory system [15]. Probably, because of the confined geographical nature and short-lived SARS-CoV-1 and MERS epidemics, the medical and scientific community did not explore other potential and secondary targets of coronaviruses. In this regard, the expansion of the current pandemic clearly showed that SARS-CoV-2 can target other systems that display ACE2 receptor. This receptor is expressed in the gastrointestinal (GI) system which makes it a potential target for the virus and can lead to GI symptoms [16]. Indeed, viral RNA particles have been detected in stool samples which raised another issue that pertains to fecal-oral transmission, be it in confined clinical and domestic settings or through sewage systems [16]. However, we do not know the prevalence and frequency of SARS-CoV-2-associated gastrointestinal manifestations in Latin America and the virus-associated symptoms and outcomes, especially in the presence of comorbidities.

There are few reports or publications made by health institutions or investigators from Latin America. Nevertheless, at this time that we are writing this paper, one of the epicenter of the disease is located in this region of the world. Taking into consideration some factors such as difficult access to healthcare in several regions of Latin America, the high index of poverty, the low availability of clean water, and waste management that further exacerbate the pandemic’s outcome [17], it would be prudent to study the most common symptoms and comorbidities to distinguish the high risk categories in order to utilize the scarce resources intelligently and wisely in every day practice in a country/population-specific manner. Here we report the results of our systematic analysis using the published and available data to date, and demonstrate that gender distribution, age range, symptoms and comorbidities are different among the 8 Latin American cohorts. Hypertension and obesity were the predominant comorbidities and diarrhea the top GI symptom.

Our study revealed that COVID-19 manifests differently in Latin America among the various regions. These differences are important to consider since their impact can influence management, treatment and prevention approaches for better outcomes when addressing the infection. In our study, the most common symptoms in Latin America are dry cough, fatigue, sore throat and fever. Also, the most common comorbidities are obesity, hypertension and diabetes [18,19]. This contrasts with what was reported by Maechler et al, from a Berlin testing center that indicates that the most common symptoms were fatigue, myalgia and cough while top comorbidities were chronic lung disease and chronic heart disease [14]. These discrepancies could emanate from the methodologies or sources of data collection in the two studies. Furthermore, our data are more recent and include larger cohorts. The conclusive evidence gathered in our systematic review can make a significant impact on screening, tagging, and separating patients into suspected and hospitalization candidate cases that are amenable for follow-ups such as contact-tracing. It is worth noting that these symptoms and comorbidities did not distribute similarly among the eight studied nations. Mexico and Chile followed a similar profile as far as comorbidities are concerned.

There is a special mention about GI symptoms in our study. The prevalence of diarrhea was much higher than reported in other investigations [20–22]. This is probably because a higher prevalence of diarrhea in Latin American populations exists prior to SARS-CoV-2 outbreak, due to the consumption of contaminated foods [23]. In our study, we examined the clinical aspects of the disease and also compared the different demographics from every population and analyzed their association with lethality, incidence and mortality. According to our research, the cumulative lethality in Latin America is 3.4%, very close to the one reported worldwide based on the current data published by John Hopkins University [7]. We also understand that the current lethality rate (a.k.a. case fatality rate) may be inflated compared to the actual risk of death because the people most likely to get tested for COVID-19 are those with severe symptoms and not those with mild symptoms or asymptomatic patients. This rate might also be deflated as many COVID-19 related deaths in Latin American countries are very likely under-reported.

Regarding the demographics of our population, there is a notable difference between sexes, females being the most affected. This is different than what Conti et al. reported on their study where males were the primary target; which they explained as an indication that women were less susceptible to viral infections due to a difference in innate immunity, steroid hormones and the presence of two X chromosomes and the immune regulatory genes associated with chromosome X [24]. Here again, we must note that underlying factors such as age, sex and pre-existing health conditions make the individual risk different from the overall risk. This is important to highlight because while certain reports of case fatality rates include some of these demographics, these numbers also have the same context-based biases as the overall lethality or case fatality rates discussed above.

Since lethality is the main outcome to avoid in this stage of the pandemic, we performed different analyses in order to compare the impact of the top comorbidities on lethality. We found that obesity, hypertension and diabetes were highly correlated with lethality, and that the presence of these conditions exacerbates the severity and outcome of COVID-19 infection. This is similar to what was reported by other studies [18,19].

Another pertinent element to highlight, is that the prevalence of tuberculosis in our study was above 1.2%. There is a well-known high prevalence of tuberculosis in the region and the usage of BCG vaccination in Latin America. Many studies reported a potential effect of the BCG vaccine in providing heterologous protection [25] against other infections like the one from SARS-CoV-2. While the BCG vaccine role is still debated, future large and well-designed comparative studies might reveal whether it played any role in the pandemic’s outcome in Latin America.

Hospital/healthcare structures and some of the observed differences between countries have to do with pandemic management and data availability. Indeed, Venezuela for example showed the least lethality, which might be explained by free access to healthcare system and the fact that the treatment protocol includes hospitalizing every positive case reported [26]. This is in contrast to other countries such as Mexico where only severe disease patients are hospitalized and less access to advanced care is available for the general population [27]. In addition, the lockdown strategies differed between countries which affected the dynamics of transmission and infection rates. The availability of medical records of the patients is very critical for the precise interpretation of the data. Some publications like the one from Chile presented complete clinical data, while the same detailed data was not available for the other regions since most of them use manual medical records and managing such data is difficult.

A limitation of our study is its retrospective nature. Some of our reports are from several months ago when the pandemic started, also we only listed studies that were available as published reports (Table 1) or on the web. Many of the health authorities in these countries have limited data sharing capability to provide solid recommendations to the healthcare sector on how to handle the pandemic. Furthermore, some of them reported merely symptoms, others only comorbidities. Therefore, there were disparities in the way they reported patients’ information. There are many variables such as immune reaction and blood test, imaging, treatment, liver enzymes and other underlying diseases that are very important for the analysis but are missing in the analyzed data. There is a need for standard collection of data for such a massive and lethal pandemic. For global standardizations, organizations such as WHO or PAHO, can recommend/provide useful tools for both electronic and manual medical records in order to collect precise data for dealing with either endemic or pandemic infections. It is relevant to comprehensively study the clinical conditions of the pandemic so each country can evaluate the most common trends in their population and get better management and treatment protocols.

GI and liver manifestations such as anorexia, dysgeusia, nausea, vomiting, diarrhea, abdominal pain, and discomfort are common among patients with COVID-19. Hepatic involvement in this condition is reflected by abnormal liver enzymes and much less frequently elevated bilirubin values. Few studies [28,29] showed that patients with the severe disease more often have GI symptoms and hepatic involvement. While the mechanisms of GI manifestations elicited by SARS-CoV-2 are still unknown, it is clear that ACE2 expression in the esophagus, small and large intestines, plays a role. Indeed, ACE2 was reported to be co-expressed with TMPRSS2 that is needed for the cleavage of the spike protein and the facilitation of the virus entry into the host GI system aggravating systemic effects of the virus and elevating the rates of transmission. While millions of patients have been discharged and tested negative in their nasopharyngeal specimens, a sizable portion of them still tested positive in their stools [30]. This group of patients should be the focus of next stringent monitoring methods as they are likely to play a role in the dissemination and recurrence of the disease. It will also be worth monitoring whether SARS CoV-2 persistence in the GI system will impact overall GI system functions.

In conclusion, while the pandemic is still raging and vaccines and therapeutics are still in development, the only direct interventions readily available now are modifications of human behavior measures such as hygiene, social distancing and mask-wearing to limit exposure and future infections. Since this disease is here to stay in the foreseeable future, strict measures need to be taken in Latin American countries to act on comorbidities such as obesity, hypertension and diabetes, all part of the metabolic syndrome. Furthermore, public health interventions to modify these comorbidities will have a positive impact on COVID-19 overall outcome in Latin America.

## Supplementary Material

Supplement

Supplement

Supplement

Supplement

Supplement

## Figures and Tables

**Table 1:** Demographics, Baseline Health and Clinical Characteristics of COVID-19 studies from Latin America

Asthma	5.2	M	M	2.9	M	4	M	1.1
**COPD**	1.8	M	4.8	1.4	M	4	2.8	M
**CV Disease**	M	M	23.7	1.5	M	3	M	M
**Chronic Cardiopathy**	2	1.8	M	1.3	M	M	1.5	0.8
**CKD**	1.2	M	1.1	1.2	M	5	1.5	0.2
**Tuberculosis**	1.3	M	M	M	M	0.1	M	M
**Immunocompromised**	1.8	M	M	1	M	3	M	M
**Cancer**	2	M	M	M	M	0.3	1	M
**Chronic Neurological Disease**	3.4	M	M	0.6	M	0.3	1.1	M
**Chronic Hepatic Disease**	0.4	M	M	0.3	M	0.8	M	M
**References**	15	16	17	18	19	Unpublished data	20	21

**Table 2:** Comparison of combined overall weighted averages for symptoms and comorbidities in selected studies

	N	Min%	Maxi%	Mean
Cough	713,555	35.5	78.8	60.1
Fatigue or tiredness	367,256	51.5	53.2	52.0
Sore throat	368,501	37.9	50.7	50.3
Fever	704,087	36.5	83.3	44.2
Headache	724,268	26.1	73.3	42.9
Myalgias	724,268	9.0	63.0	33.9
Odynophagia	346,299	27.0	45.9	29.9
Nasal congestion	360,791	0	24.1	23.8
Dyspnea	703,980	4.3	47.6	22.8
Hypertension	718,814	1.8	28.8	12.1
Diarrhea	703,980	3.1	22.9	11.5
Thoracic pain	703,980	2.2	31.9	9.2
Anosmia	687,412	1.400	36.1	8.8
Diabetes	708,101	0.6	20.6	8.3
Nausea and Vomiting	397,540	1.2	7.9	7.2
Ageusia	724,161	0.4	37.1	7.0
Obesity	714,800	3.9	25.6	4.5
Abdominal pain	703,980	1.5	16.6	4.3
Joint pain	397,540	3.2	59.0	3.6
Asthma	361,026	1.1	5.6	3.2
Tachypnea	346,299	3.0	4.0	3.0
COPD	714,693	1.2	4.0	2.1
Cyanosis	320,263	0.6	29.7	1.6
CV Disease	309,550	1.5	3.1	1.5
Chronic Cardiopathy	715,704	0.8	4.8	1.5
CKD	707,994	0.2	4.9	1.3
Tuberculosis	39,859	0.1	1.3	1.2
Immunocompromised	346,299	1.0	2.9	1.1
Cancer	397,540	0.3	2.0	1.0
Chronic neurological disease	703,980	0.3	3.4	1.0
Chronic hepatic disease	346,299	0.3	0.80	0.3

N = Number of patients that was used to analyze. Minimum and Maximum % = means the minimum/maximum average value that has been reported in this multi-center study by included reports. For example, minimum age means the smallest average age that has been reported and maximum age means the largest average age that has been reported.

## Abbreviations

ACE2: Angiotensin Receptor 2
CI: Confidence Interval
COVID-19: Coronavirus Disease-19
GI: Gastrointestinal
RT-PCR: Real-time Polymerase Chain Reaction
SARS-CoV-2: Severe Acute Respiratory Syndrome Coronavirus 2
TMPRSS: Transmembrane Serine Protease
