# Supplementary material for: A Comprehensive Analysis of COVID-19 Impact in Latin America
Source: Res Sq. 2021 Jan 8:rs.3.rs-141245. Preprint. [Version 1] doi: 10.21203/rs.3.rs-141245/v1 (PMC7805457; doi:10.21203/rs.3.rs-141245/v1)
Supplement: Supplement [file 36c7bd7b47cb09c6850dfbbd.docx]

Supplemental materials:

Table 1 Suppl. Comparison of combined overall weighted average for lethality rate, average age, male and female rates in selected studies from Latin American countries.

| N=728,282 | Min % | Maxi % | Overall % | Std. Deviation |
| --- | --- | --- | --- | --- |
| Lethality Rate | 0.947 | 16.7 | 3.4 | 1.14 |
| Average Age | 39 | 56.8 | 48.4 | 8.78 |
| Male | 43.2 | 56.7 | 47.3 | 4.29 |
| Female | 43.3 | 56.8 | 52.6 | 4.29 |

N=number of patients that was used to analyze. Minimum and Maximum % = means the minimum/maximum average value that has been reported in this systematic review by included reports. For example, minimum age means the smallest average age that has been reported and maximum age means the largest average age that has been reported.
