# Supplementary figures and images for: A Comprehensive Analysis of COVID-19 Impact in Latin America
Source: Res Sq. 2021 Jan 8:rs.3.rs-141245. Preprint. [Version 1] doi: 10.21203/rs.3.rs-141245/v1 (PMC7805457; doi:10.21203/rs.3.rs-141245/v1)

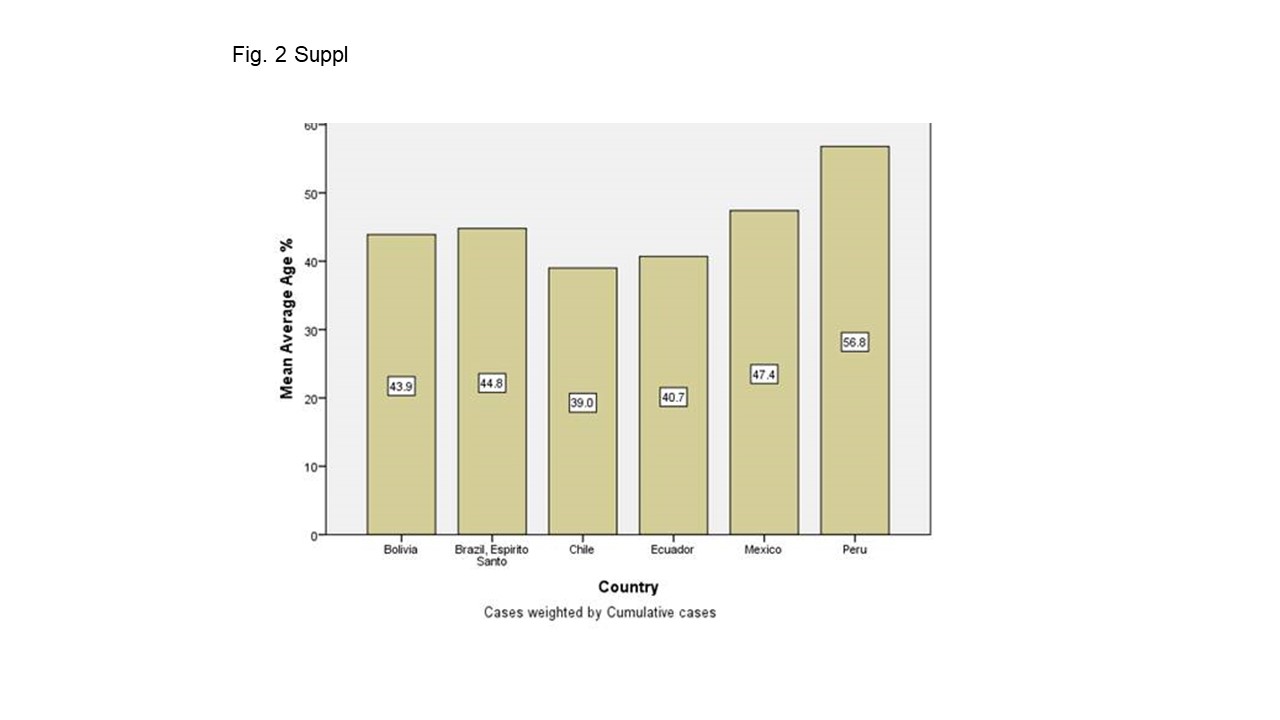

Supplement: Supplement [file 4e144db9b5d15dd28ac453cd.jpg]

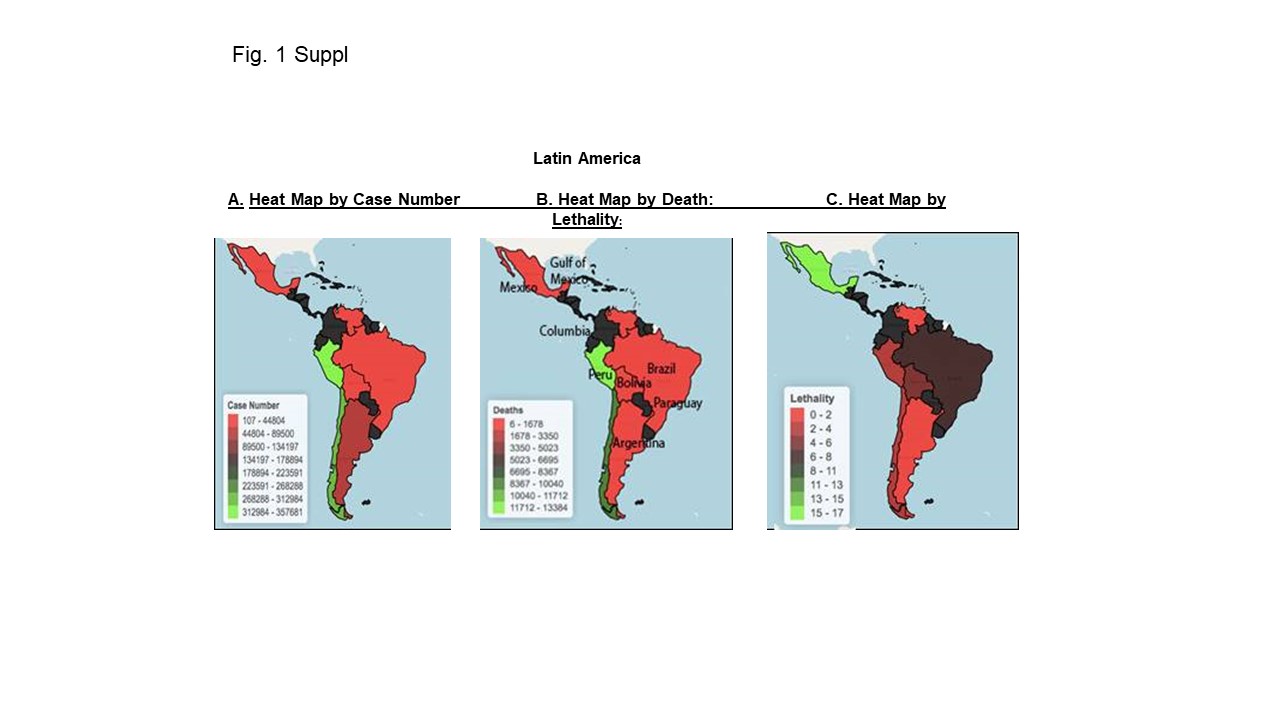

Supplement: Supplement [file 5c33e21378854aa49d4fad93.jpg]

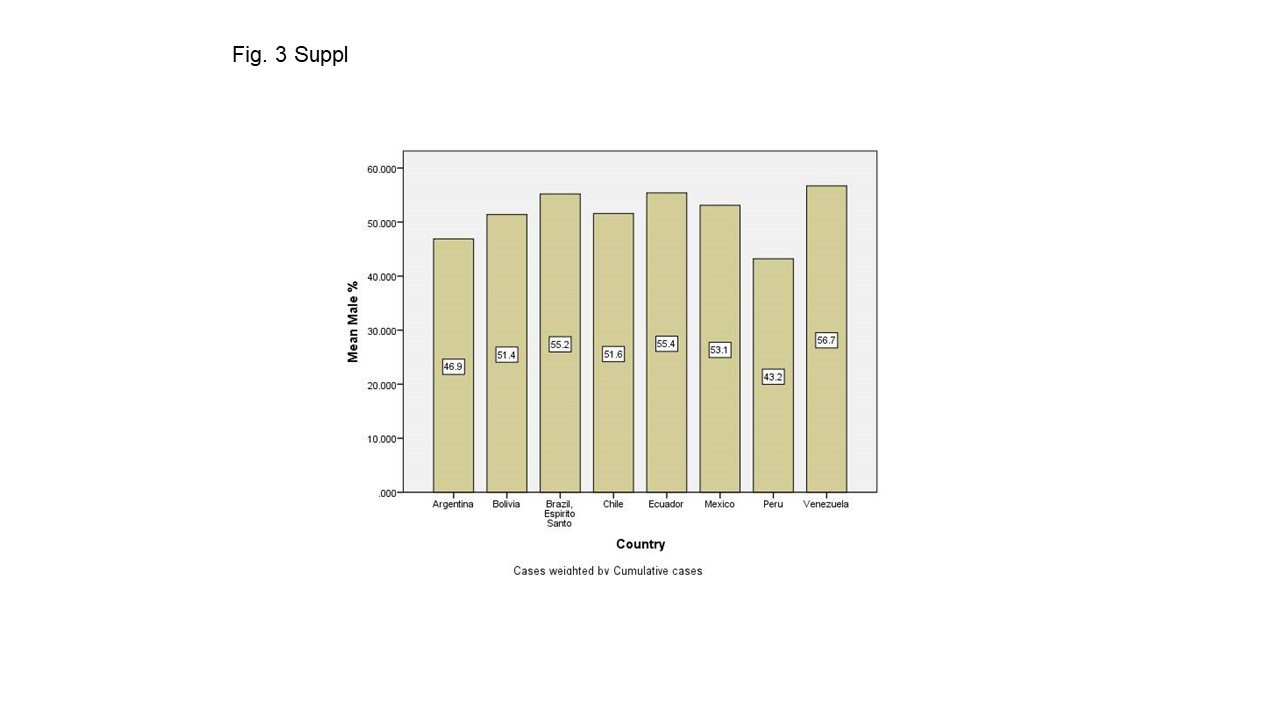

Supplement: Supplement [file d14fc17698035a299e75aaa8.jpg]
