# Supplementary material for: A Comprehensive Analysis of COVID-19 Impact in Latin America
Source: Res Sq. 2021 Jan 8:rs.3.rs-141245. Preprint. [Version 1] doi: 10.21203/rs.3.rs-141245/v1 (PMC7805457; doi:10.21203/rs.3.rs-141245/v1)
Supplement: Supplement [file af0363a60de773235cd438ae.docx]

Supplemental materials:

Table 2 Suppl. Analysis to assess the relationship between lethality (dependent variable) and Hypertension, Obesity, Diabetes and Asthma (Independent Variables).

|  | | | | | |
| --- | --- | --- | --- | --- | --- |
| Comorbidity | Unstandardized Coefficients | | Standardized Coefficients | t | Sig. |
|  | B | Std. Error | Beta |  |  |
| Hypertension | .522 | .154 | .834 | 3.376 | .020 |
| Obesity | .437 | .131 | .857 | 3.322 | .029 |
| Diabetes | .770 | .170 | .914 | 4.518 | .011 |
| Asthma | 1.264 | 1.853 | N/A | .366 | .682 |
|  | | | | | |

In multivariable analyses, the leading and significant comorbidities were diabetes (β =0.77, p=0.01), obesity (β =0.44, p=0.03), and hypertension (β=0.522, p=0.02). However, asthma (β =1.26, p=0.68) was not associated with higher mortality.
